# Bypassing health facilities in rural Mozambique: spatial, institutional, and individual determinants

**DOI:** 10.1186/s12913-018-3834-y

**Published:** 2018-12-29

**Authors:** Jing Yao, Victor Agadjanian

**Affiliations:** 1 Urban Big Data Centre, School of Social and Political Sciences, University of Glasgow, 7 Lilybank Gardens, Glasgow, G12 8RZ UK; 2 Department of Sociology, University of California, Los Angeles, Los Angeles, CA 90095-1551 USA

**Keywords:** Hospital bypass, Healthcare utilization, GIS

## Abstract

**Background:**

Access to sexual and reproductive health (SRH) services is critical for such outcomes as pregnancy and birth, prenatal and neonatal mortality, maternal morbidity and mortality, and prevention of vertical transmission of infections like HIV. Health facilities are typically set up where they can efficiently serve the nearby targeted population. However, the actual utilization of health care can be complicated as people sometimes bypass the closest or nearby facilities for various reasons such as service quality. A better understanding of how people actually utilize health services can benefit future health resource allocation as well as health program planning.

**Methods:**

In this study, we use prenatal care as an example of a basic, widely available service to investigate women’s choice and bypassing of SRH facilities as well as potential influencing factors at the geographic, clinic, household, and individual levels. The data come from a population-based survey of women of reproductive age in rural Mozambique. The spatial pattern of utilization of health clinics for prenatal care is explored by geographical information system (GIS)-based spatial analysis. Logistic regression is fitted to test the hypotheses regarding the effect of distance, service quality, and household/individual-level factors on the bypassing of the nearest clinic.

**Results:**

The results indicate that most women living near clinics tend to utilize the closest facilities for prenatal care and those who travel farther mainly do so to seek better services. Further, for women who live far from a clinic (> 5.5 *km*), service quality still plays an important role in the facility bypassing while the effect of distance is no longer significant. The bypassing of nearest facility is also affected by individual characteristics such as age, HIV status, and household economic conditions.

**Conclusions:**

The findings help to better understand health facility choice and bypassing in developing settings, in general, and in resource-limited Sub-Saharan settings, in particular. They offer valuable guidance for future health resource allocation and health service planning.

## Background

Access to and utilization of health services have long been a great concern to both policy makers and general public as they critically shape health outcomes and overall wellbeing. Access is a multifaceted concept and can be viewed from different perspectives such as social, organizational and geographical [1–3]. Accordingly, access has been defined in the literature using population-to-physician ratio, travel distance/time, or gravity model-based measures combining various factors [4]. Utilization is usually thought of as “realized access” [1]. Importantly, potential access to health facilities does not necessarily ensure sufficient utilization of services that they offer because healthcare seeking and use are complex behaviours usually involving a variety of individual, socioeconomic, institutional and spatial components [5–7]. A better understanding of how people actually utilize health services can not only help access the validity of perceived access to such services but also assist with future health resource allocation and health program planning.

One way to understand health service utilization is to examine health facility choice and the bypassing behaviour, which can shed light on which facilities are underused, as well as the characteristics of individuals and facilities affecting the decision-making regarding healthcare usage [6–9]. In particular, bypassing the nearest or nearby facility has received extensive attention, as it is said to lead to the underutilization, financial strain or even closure of some hospitals especially in rural areas [8, 10–12]. It has also been argued that factors contributing to the bypassing of health facilities vary across different healthcare systems, regions, population groups and individuals. Yet, the impact of some factors, such as geographic access, service quality and cost, age and income, has been consistently shown in numerous empirical studies [6, 7, 9, 13–15].

Most existing studies, however, have focused on the bypassing of health facilities in developed countries such as the US [6, 9, 10, 14], the UK (e.g. [7]) and France (e.g. [16]). Relatively little is known with regard to whether, how and why people bypass local facilities in developing, especially resource-limited settings, such as those of Sub-Saharan Africa, where inefficient usage of existing health facilities can greatly affect the effectiveness of public health services that are often already strained due to chronic shortages of medicines and qualified staff. In such settings, it is particularly crucial that existing healthcare resources are efficiently utilized and the bypassing of facilities for standard services is avoided as much as possible [15].

The aim of this study is to examine women’s bypassing of the nearest sexual and reproductive health (SRH) facility in a resource-poor rural setting in Mozambique and to investigate the underlying driving factors, particularly distance and service quality as well household- and individual-level characteristics. Access to and utilization of SRH services in such settings are highly consequential for a variety of health outcomes such as pregnancy and birth, prenatal and neonatal mortality, maternal morbidity and mortality, and vertical transmission of infectious diseases like HIV/AIDS. Distance and service quality have been found closely related to SRH service utilization in Sub-Saharan Africa [17, 18]; yet, how and to what extent they affect the bypassing of the nearest facility remains underexplored. The particular SRH service of interest here is prenatal consultation which is available free of charge at every local clinic in the study area. Given the limited health resources and poor transportation networks in rural Mozambique, it is reasonable to expect women to visit the nearest clinic for such a standard SHR service. The focus of this analysis is on when and why this might not be the case.

The remainder of the paper is structured as follows. The next section reviews the existing evidence on the factors associated with hospital choice and bypassing, based on which the research hypotheses are formulated. It is followed by a description of the study setting and of the data and research methods. Then, the results of the analysis are presented. The paper concludes with a summary of main findings and a discussion of their implications for health program planning and management.

## Research hypotheses

The choice and bypassing of health facilities can be linked to theories of human geography. First, as suggested by the distance decay effect – a common law for human activities in geographic space -- the utilization of health facilities decreases with increased travel distance. Further, a fundamental concept of the central place theory [19] -- the “range”, when adapted to the purpose of this research, implies that there is a maximum distance that patients are willing to travel to acquire health services. Again, according to the urban hierarchy argument of the same theory, whereby larger centres have greater market area covering both their own and satellite settlements, urban hospitals function to serve the surrounding rural areas in addition to urban residents. Finally, the outshopping theory, which suggests that people might favour farther away retailers for multiple goods or better deals [20], implies that patients might bypass local facilities to seek more or better health services at more distant health units [9].

Studies have examined various characteristics of facilities and individuals, as well as the interactions between them, that influence bypassing [7, 9]. Commonly identified relevant facility characteristics include size, service quality, type, cost, and ownership (e.g. public or private), among others [6, 13–15]. For example, it was found in the United States (U.S.) that hospitals of larger size and more services are usually more attractive [6, 11, 21]. Roh et al. [14] showed that women in rural Colorado in the U.S. preferred private over public hospitals and were more likely to visit networked hospitals for obstetric services. In a study in England, patients preferred a hospital with less waiting time or better quality even if it was located farther away [7]. For developing countries, quality of services was found to be an important determinant of clinic bypassing net of other factors in Sri Lanka [13], India [15], and Tanzania [22].

Relevant individual characteristics identified in the literature include insurance status, age, education, socioeconomic status, personal medical conditions or the complexity of illness, etc. [6, 10, 11, 21]. Much evidence with respect to the impact of personal characteristics on health-seeking behaviour has been produced in the U.S. context. For example, patients with medical, especially commercial insurance tend to bypass local rural hospitals despite the availability of desired treatment [11, 14]. While most rural older beneficiaries tend to use the nearest hospitals [8, 10], some might favour urban hospitals if they need in-patient services [21]. Particularly, for child deliveries, Alford-Teaster et al. [12] found that in the U.S. most rural women who bypassed the nearest mammography facilities lived near an urban area and were from more affluent communities. In addition, it was found that in both developed [23] and developing [16, 22, 24] settings service quality of maternity hospitals is a main choice criterion for women of both low- and high-risk.

Distance, or geographical access in general, is also a primary concern in healthcare choice, especially for the population groups with limited mobility, such as rural residents or the elderly [11, 16]. It was found in the U.S. that decreased travel distance would increase the likelihood of health facility use and therefore reduce the probability of bypassing [21]. The role of distance in health care utilization may be particularly important in resource-limited settings such as rural sub-Saharan Africa [18, 25, 26].

In sum, although most evidence on healthcare seeking choice and behaviours comes from developed settings, some of its key determinants, such as economic status, education level, distance and quality of service, are not unique to those settings and are applicable, with appropriate caveats, to developing contexts like those in sub-Saharan Africa. Therefore, adapting the reviewed cross-national evidence to the societal and healthcare context of rural Mozambique, we formulate and test the following five main hypotheses regarding why some women may bypass the nearest clinics for prenatal consultations:

- Hypothesis 1: The farther the nearest clinic is from a woman’s residence, the more likely she is to bypass that clinic for prenatal care in favour of other clinics.
- Hypothesis 2: Women are more likely to bypass the nearest clinic for prenatal care if that clinic offers fewer/lower-quality services.
- Hypothesis 3: Women from more affluent households are more likely to bypass the nearest clinic for prenatal care.
- Hypothesis 4: Women with higher educational levels are more likely to bypass the nearest clinic for prenatal care.
- Hypothesis 5: Women with heightened health risks, such as advanced age or HIV positive status, are more likely to bypass the nearest clinic for prenatal care.

In addition, in order to investigate the relationship between distance and service quality based on the concept “range” of the central place theory, we test the following two hypotheses:

- Hypothesis 6: Clinic service quality does not affect bypassing for women whose actual travel distance to clinic for prenatal care is less than the median “range”, but does affect bypassing for women whose actual travel distance to clinic for prenatal care is larger than the “range.”
- Hypothesis 7: Distance to the nearest clinic does not affect bypassing for women whose actual travel distance to clinic for prenatal care is larger than the median “range”, but does affect bypassing for women whose actual travel distance to clinic for prenatal care is less than the “range.”

## Methods

### Data

The data for this study were collected in rural areas of four contiguous districts in Gaza province in southern Mozambique. The four districts, Chibuto, Chókwè, Guijá and Mandlakaze, have a total area of approximately 16 thousand *km*^*2*^ with a population estimated at about 620 thousand as of the 2007 Census [27]. This area is traditionally patrilineal and largely monoethnic. The mainstay of the local economy is subsistence agriculture, but the area is also characterized by large male labour out-migration, primarily to neighbouring South Africa. With adult HIV prevalence of about 25%, Gaza province has the highest level of HIV infection among all Mozambique’s provinces [28]. Health needs of the local population are served by a network of state-run clinics that provide basic SRH services, including prenatal care, free of charge. Although people are expected to use nearest facilities for these services, there are no formal restrictions on the choice of different clinics even in the absence of specific referrals.

The data come from Wave 3 of a longitudinal project focused on health and wellbeing of rural women and their families. Wave 1, conducted in 2006, a survey was carried out with 1680 married women aged 18–40 randomly sampled in 56 villages of the four districts (14 villages in each district). About 78% of those women were re-interviewed in 2009 (Wave 2) and 74% in 2011 (Wave 3). A refresher sample was added in these two waves to compensate for attrition due to respondents’ death or unavailability. In all the waves, the survey had a participation rate of nearly hundred percent. The Wave 3 questionnaire covered a wide range of sociodemographic and health-related characteristics; women were also asked in which clinic they had most prenatal consultations before their last childbirth (nearly all respondents received at least one prenatal consultation). Excluding respondents who did not have a birth or who visited clinics located far away (> 20 *km*) from the study area because they were staying outside their villages during pregnancy, the analytic sample consists of 1823 women. In parallel with the survey of women, detailed information on all local clinics was also collected through a health facility survey. All those clinics provide basic SRH services such as prenatal and postnatal care, child delivery, and family planning, but some larger clinics also offer additional services. There is a total of 62 health clinics included in this study, among which 59 are inside the study area (the four districts) and 3 are within 5 *km* of it, allowing for the fact that the nearest facility might be in a different administrative area. The clinics are ranked from 1 to 4 with higher values indicating better service quality measured by the nature and variety of available services. The rank measure is constructed using a weight-sum approach involving a range of attributes in relation to health service quality derived from the clinic survey, such as the number of nurses and rooms, and whether the clinic has received aid from any non-governmental organization. Although the information on which this measure is based does not capture all the unique details of the clinics’ everyday functioning, it is important to note that because all of the clinics are state-run, they all are subject to standard rules regarding the schedule and timing of services and other related institutional regulations (see [29] for a detailed description of this measure). Based on this ranking, 15, 36, 6, and 5 clinics are assigned ranks 1, 2, 3, and 4, respectively.

### Spatial and statistical analysis

To test the hypotheses about women’s bypassing of the nearest clinic, our study uses a combination of geographical information system (GIS)-based spatial analysis and multivariate regression techniques. First, descriptive statistics on individual characteristics and prenatal care use are generated. Then, the spatial pattern of utilization of local health facilities for prenatal care is explored by desktop mapping and exploratory spatial analysis. Finally, logistic regression is fitted to test the hypotheses regarding the effect of distance, service quality, and household/individual-level factors on the bypassing of the nearest clinic.

Given the importance of geographical context in healthcare access and utilization, GIS and exploratory spatial analysis has been widely applied in health research with a range of techniques such as visualization and spatial statistics [3, 4]. In this study, women’s choice and bypassing of nearest clinic is examined from two perspectives – village and clinic. For each village, two statistics are produced: the proportion of respondents who (1) visited each clinic and (2) bypassed the nearest clinic (bypassing rate). To explore the utilization of the clinics based on proximity, a Voronoi diagram is generated by using the clinics as seeds, which divides the study area into a set of sub-areas so that each clinic is closer to the respondents within its sub-areas than the other clinics. The partitioning of sub-areas is based on Euclidean distance as it remains a valid proxy for geographic access in impoverished rural settings where travel largely relies on walking or public transportation and there is a lack of actual travel data or where self-reported travel time may be highly inaccurate [18, 30]. Also, it is reasonable to assume that women will visit the nearest clinic as the standard package of prenatal care is freely available in all clinics. For each clinic, a utilization rate is defined as the ratio of the number of respondents that actually visited it to the total number of respondents within its sub-area (i.e. the expected number of users), which will have a value 1 if all the respondents visit the nearest clinic, > 1 if more women visit that clinic than expected, and < 1 if less women visit that clinic than expected.

Compared to the spatial analysis which includes all 1823 respondents, the logistic regression analysis is based on 1710 respondents who visited up to the fifth nearest clinic, thus excluding the extreme cases when respondents visited the clinics located so far away from the residence that their choice was unlikely driven by the common factors identified above. Although bypassing can be defined differently in different contexts (e.g. rural vs. urban, see [9]), of interest here is whether women bypassed the nearest clinic. Thus, the dependent variable is whether the respondent used a clinic other than the nearest one; it takes the value of 1 if she did and 0 if she visited the nearest clinic.

Reflecting the hypotheses, the predictors of interest are distance from residence to the nearest clinic; service quality of the nearest clinic; household material assets (a 1–5 scale based on household ownership of such items as radio, TV set, bicycle, motorcycle, and automobile, with higher values indicating more assets); respondent’s education (years of completed schooling); and respondent’s age and self-reported HIV status (a binary variable that takes the value of 1 if she reported being certainly or very likely HIV positive, and 0 if otherwise otherwise). The following characteristics that have been found to be related to healthcare utilization, particularly in resource-limited rural African settings [18, 25, 30], are included as controls: self-rated health (1 if considered herself to be in good health and 0 otherwise), lifetime number of pregnancies, work outside subsistence agriculture (1 if currently working and 0 otherwise), and religious affiliation (1 if affiliated with organized religion and 0 if otherwise). The analyses also control for current marital status. Given the large-scale male temporary labour out-migration from the study area and potential implications of both migrants’ remittances and their physical absence for their wives’ health care utilization, we distinguish between respondents married to migrants and those married to non-migrants. Hence, marital status is a set of dummy variables: married to migrant, married to non-migrant, and not married. Finally, the number of nearby clinics (within 10 *km*) is controlled for as an indicator of the availability of health services.

## Results

Table 1 describes the demographic profile of the respondents and the geographic access to and availability of local health services. As can be seen, about 30.8% respondents bypassed the nearest clinic for prenatal care. Although the average distance to the nearest clinic is about 4.6 *km*, some women need to travel up to 18.4 *km* to receive prenatal care. The average age of the respondents is about 33 years and the mean educational attainment is 3 years of schooling. In terms of economic characteristics, most respondents do not engage in any activities other than subsistence agriculture (65.2%) and are largely from poor households (43.8% with the asset score of 0 compared to only 9.3% with the score of 5). Three-fourths of respondents (74.9%) consider themselves being in good health, and one-tenth (9.7%) said they are certainly or likely HIV positive. Most women are in marital unions (88.9%), with about one-third (31.3%) married to migrants. Most of them had several pregnancies, with the maximum as high as 13. The overwhelming majority have a religious affiliation (usually with a Christian church). In terms of the availability of health services, almost one-half of respondents (47.8%) respondents do not have any clinic within 5 *km* of their residence, and 7.9% respondents need to travel more than 10 *km* to reach a clinic.

**Table 1:** Descriptive statistics of the survey respondents

Variable	*N* (percent)	Range (Min, Max)	Mean	Standard Deviation
Outcome				
Women bypassed the nearest clinic for prenatal care	560 (30.8)			
Predictors				
Distance to the nearest clinic (*m*)		(68.1, 18,370.6)	4588.7	3474.7
Rank of the nearest clinic		(1, 4)	2.1	0.8
Assets				
1	797 (43.8)			
2	432 (23.7)			
3	173 (9.5)			
4	249 (13.7)			
5	170 (9.3)			
Years of education		(0, 12)	3.0	2.4
Age		(21, 55)	32.6	6.3
Certainly or likely HIV positive (self-reported)	176 (9.7)			
Controls				
In good health (self-reported)	1364 (74.9)			
Married	1618 (88.9)			
To a migrant	570 (31.3)			
To a non-migrant	1048 (57.6)			
Lifetime number of pregnancies		(1, 13)	4.6	2.0
Work outside subsistence agriculture	633 (34.8)			
Having a religion affiliation	1702 (93.5)			
Number of clinics within 10 *km*		(0, 7)	2.3	1.5

Sample size: 1821

The utilization of prenatal care is summarized in Table 2, where the respondents are grouped into six categories based on the closeness of the visited clinics. Within each category, the ranks of the nearest clinic and the visited clinic are compared based on the proportion of the respondents in that category. As can be seen, about four-fifths of respondents (79.7%) sought prenatal care in either the nearest (69.2%) or the second nearest (10.5%) clinics to their residences and only 6.1% respondents visited a clinic located farther than the fifth closest clinic, which supports the assumption that most women would utilize the nearest clinic. Respondents who did not visit the closest clinics tended to choose the clinics with better services, which was particularly true for those who travelled further than the second closest clinic. For example, among all the respondents who visited the fifth closest clinic, about 82.1% selected a clinic offering better services than their closest clinic. This supports our hypothesis that service quality would be associated with the bypassing of the nearest clinic. Regarding the distance to the nearest clinic for each group, it has the smallest value on average, about 4.1 *km* for the respondents who visited the closest clinics. In fact, the respondents who did not use the closest prenatal care are on average 5.7 *km* away from the nearest clinic although its value varies across different groups of respondents as shown in Table 2, which might be attributed to other characteristics. Nevertheless, this indicates that distance to the nearest facility is related to bypassing behaviour.

**Table 2:** Clinic choice of the survey respondents

Visited Clinics	% of Respondents	Rank of Clinics (%)^a^			Distance to the Nearest Clinic (*km*)			Extra Distance to Visited Clinic from Nearest Clinic (*km*)		
		V < N^#^	V = N	V > N	Range (Min, Max)	Mean	Standard Deviation	Range (Min, Max)	Mean	Standard Deviation
Closest	69.2				(0.1, 17.2)	4.1	3.3			
2nd closest	10.5	21.4	48.4	30.2	(0.1, 12.8)	5.6	3.2	(0.1, 24.8)	3.3	3.8
3rd closest	7.2	3.1	32.1	64.9	(0.3, 18.4)	6.1	3.5	(0.4, 35.1)	8.8	5.8
4th closest	3.8	4.3	25.7	70.0	(0.4, 10.0)	6.3	3.1	(1.2, 19.7)	9.9	5.5
5th closest	3.1	1.8	16.1	82.1	(0.6, 9.9)	4.5	3.0	(2.6, 15.4)	8.8	3.4
Other	6.1	3.6	7.2	89.2	(0.1, 14.7)	5.6	4.6	(4.7, 119.3)	21.2	19.8

Sample size: 1821

^a^(% is calculated within each category)

#: V: visited; N: Nearest

**Fig. 1:**
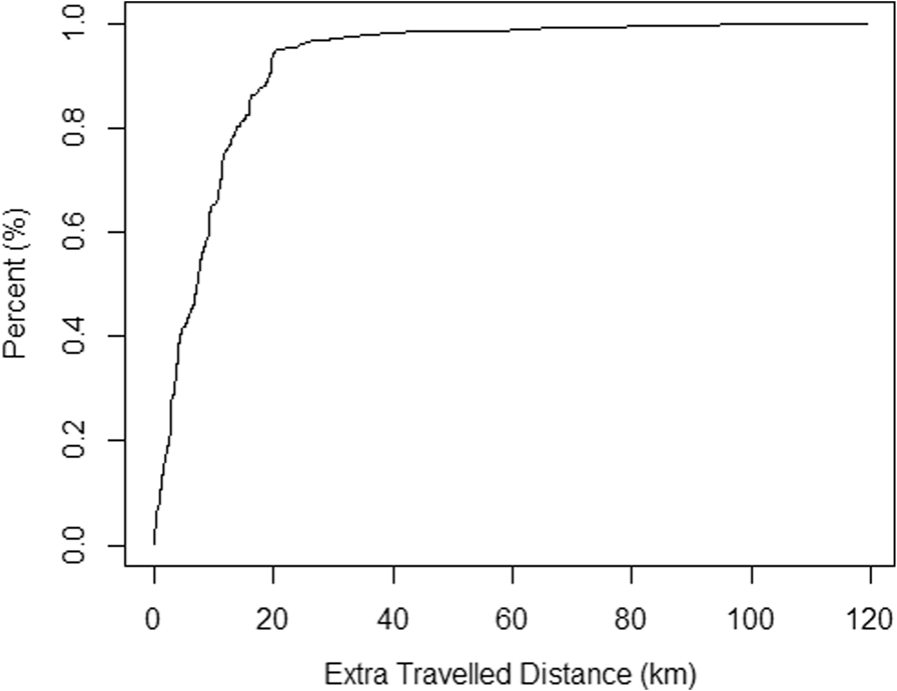
Distribution of extra travelled distance in seeking prenatal care

The distribution of extra distance that women travelled to seek prenatal care is provided in Fig. 1. On average, women travelled extra 9.5 *km* (i.e., beyond the distance to the nearest clinic) to receive prenatal care; among them about 41.8% travelled up to 5 *km* extra*,* 64.8% up to 10 km, and 93.7% up to 20 *km*. Yet, extra travel distance varies across different group of respondents, as shown in Table 2. For example, respondents who visited the second closest clinic have the lowest value of extra travel, about 3.3 *km*.

**Fig. 2:**
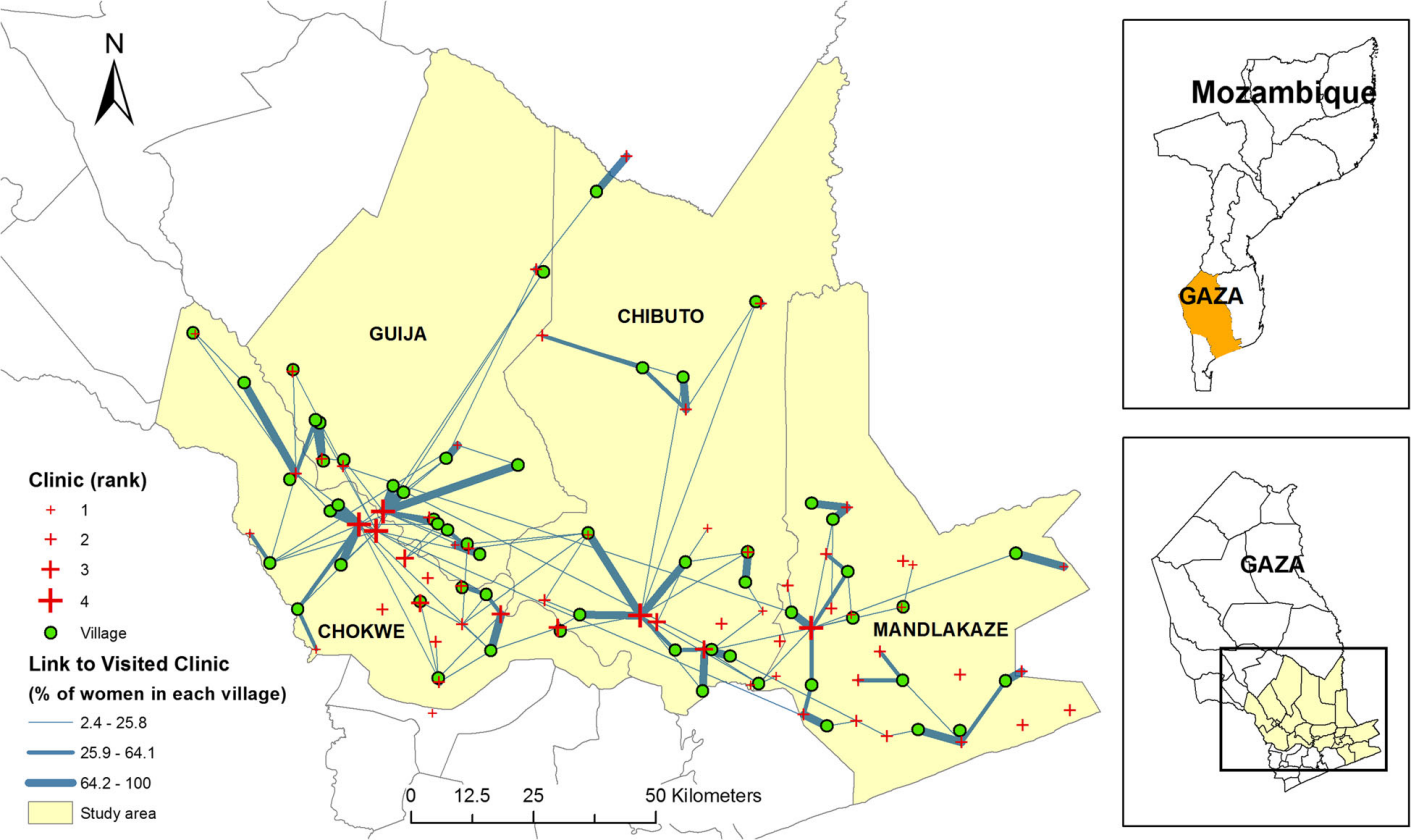
Spatial distribution of clinic choice for each village

The spatial pattern of clinic choice and bypassing is depicted in Fig. 2. The dots represent the location of villages (based on averaged coordinates of residences of respondents in each village), and the crosses represent the clinics with larger size indicating better service quality. Villages and clinics are connected by straight lines with the width of the lines indicating the proportion of women from each village that used each clinic, and three types of line are differentiated by classifying the proportions using Jenks natural breaks. It can be observed that most women from the five villages in the north, where the population is sparser and clinics are fewer, used the nearest or nearby clinics. In contrast, the pattern of clinic choice and bypassing is more complicated in the south where most respondents and clinics are located. This implies that women tend to use the closest clinic when there are fewer clinics available within certain distance from the residence (i.e. in the north of the study area), which is the opposite for the women in the south. Further, as the graph suggests, the five highest-ranked clinics (located mostly in district capitals) attracted many respondents from the nearby rural areas for whom they are not necessarily the closest health units, which again indicates the importance of service quality in clinic choice.

**Fig. 3:**
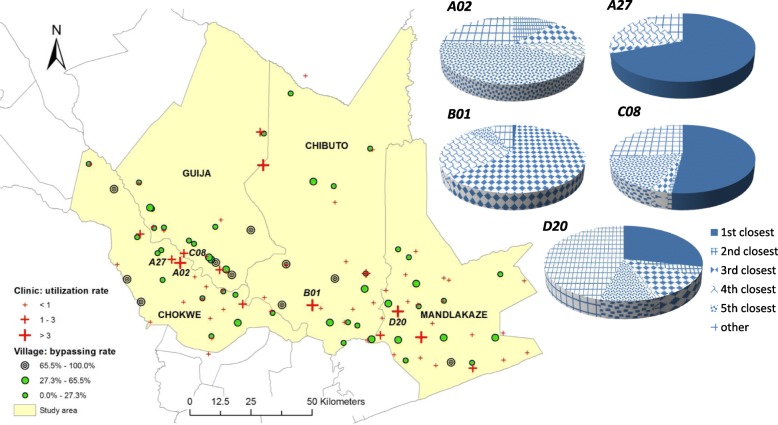
Spatial variations in clinic utilization rate and village bypassing rate

The utilization and bypassing of clinics is further explored by the utilization and bypassing rates, as shown in Fig. 3. Three-fourths (76.3%) of the clinics within the study area have a utilization rate of less than 1. Five clinics have a utilization rate higher than 3, among which three are district-level clinics (ranked 4) in Chókwè (coded *A02*), Chibuto (*B01*) and Mandlakaze (*D20*), respectively, again pointing to the importance of service; the other two are ranked 2, one on the border of Guijà and Chibuto and the other located in Mandlakaze. Both of the other two district clinics (*A27* and *C08*) that are close to the border between Chókwè and Guijà have a utilization rate between 1 and 3, which is possibly affected by the proximity of another district clinic, *A02*. The utilization of prenatal services in the district clinics is further examined by their proximity to respondents’ residences, as shown by the pie charts included in Fig. 3. It can be observed that two district clinics, *A02* and *B01*, were largely utilized as a service provider beyond the second closest clinic. In contrast, more than half of the patients used *A27* (69.4%) and *C08* (52.0%) as the nearest service provider, which might reflect both distance and service quality given the existence of several villages around those two district clinics as shown in both Figs. 2 and 3. All five clinics received patients from more distant villages (i.e., they were used as the clinics located farther than the fifth closest), with the share of such patients varying from 6.2% (*B01*) to 45.7% (*D20*).

Similarly, the village-level bypassing rate also varies across space. In total, ten villages have a bypassing rate higher than 65.5%; all of those villages are located in the south with one in Mandlakaze and three in each of the other districts. Considering the clinic choice pattern displayed in Fig. 2, it seems that most respondents from those ten villages visited the corresponding regional clinics within their districts. The exceptions are two villages, one in northern Chókwè and another in Mandlakaze, where respondents visited the nearby clinics offering similar or better services. All those observations suggest that service quality might be an essential concern in prenatal care choice, which, along with distance and other covariates, are further examined through the regression analysis as detailed below.

The results of confirmatory logistic regression analysis testing the study hypotheses are presented in Table 3 as odds ratios and the confidence intervals. Odds ratios above unity indicate a positive effect on the likelihood of bypassing the nearest clinic; odds ratios below unity indicate a negative effect. First, as can be seen, distance is a significant predictor of the bypassing behaviour, that is, for every 1 *km* increase in the distance to the nearest clinic, the odds of bypassing that clinic will increase by .23, ceteris paribus, which supports Hypothesis 1. Similarly, service quality is a significant predictor: compared to having the lowest ranked clinic as the nearest clinic, having the nearest clinic ranked 2, 3, or 4 decreases the odds of bypassing by 62, 78, and 93%, respectively, net of other factors. Hypothesis 2 is therefore also confirmed. Our hypothesis regarding household wealth (Hypothesis 3) is also generally supported. Greater wealth increased the probability of bypassing the nearest clinic; interestingly, however, only women living in most affluent households (with assets scores of 4 and 5) were significantly more likely to bypass the nearest clinic. At the same time, contrary to what was predicted in Hypothesis 4, education had no effect on clinic bypassing. Finally, for Hypothesis 5, both age and HIV status have a significant impact on clinic bypassing in the predicted direction. Specifically, for every 1 year increase in age, the odds of bypassing the nearest clinic increase by 2% while controlling for all the other factors. Among those who were certainly or likely HIV positive, the odds of bypassing the nearest clinic are 68% higher than among the rest.

**Table 3:** Logistic regression results of clinic bypassing

Variable	Odds Ratio	95% Confidence Interval
Intercept	0.12	(0.04, 0.34)^a^
Distance to the nearest clinic	1.23	(1.18, 1.29)^a^
Rank of nearest clinic (vs. 1)		
2	0.38	(0.29, 0.50)^a^
3	0.22	(0.15, 0.34)^a^
4	0.07	(0.04, 0.11)^a^
Assets (vs. score 1)		
score 2	0.86	(0.63, 1.18)
score 3	0.77	(0.50, 1.18)
score 4	1.50	(1.05, 2.13)^a^
score 5	1.44	(0.94, 2.17)^a^
Years of education	1.02	(0.97, 1.07)
Age	1.02	(1.00, 1.05)^a^
Certainly or likely HIV positive (self-reported)	1.68	(1.13, 2.46)^a^
In good health (self-reported)	0.85	(0.64, 1.12)
Married (vs. unmarried)		
To a migrant	1.18	(0.77, 1.83)
To a non-migrant	1.29	(0.87, 1.95)
Lifetime number of pregnancies	0.96	(0.89, 1.03)
Work outside subsistence agriculture	0.89	(0.69, 1.14)
Has a religion affiliation	0.91	(0.57, 1.49)
Number of clinics within 10 *km*	1.22	(1.10, 1.35)^a^

*N* = 1710

^a^significant at 0.05 level

Among other covariates, the number of clinics within 10 *km* has a significant positive effect on the likelihood of bypassing the nearest clinic. No individual-level controls show an effect that reaches the threshold of statistical significance.

In order to test our Hypotheses 6 and 7 about possible variation in the effect of service quality and distance to the nearest clinic by actual travel distance, we first define the median distance (about 5.5 *km*) that all the respondents travelled to receive prenatal care as the “range”, based on which the respondents are divided into two groups: Group 1, with travel distance smaller than 5.5 *km*, and Group 2, with travel distance greater than 5.5 *km*. Then the same regression analysis as in Table 3 is implemented for each group of respondents separately. The results are presented in Table 4. As can be seen, service quality remains a significant predictor for both groups of women, which does not support our expectation in Hypothesis 6 that it would not matter for women travelling shorter distances to obtain services. At the same time, the distance to the nearest clinics still a significant predictor for Group 1 but not for Group 2 (the value 1.00 for both odds ratios and confidence intervals are the value rounded to two digits), indicating its lesser importance compared to service quality for the women who travelled longer distances. Thus Hypothesis 7 is supported. Also, compared with the results in Table 3, HIV infection is no longer a significant predictor for Group1. For Group 2, age is not significant.

**Table 4:** Logistic regression results of clinic bypassing for two sub-groups of respondents based on travel distance

Variable	Group 1 (sample size: 1007) (travel distance < = 5.5 *km*)		Group 2 (sample size: 703) (travel distance > 5.5 *km*)	
	Odds Ratio	95% Confidence Interval	Odds Ratio	95% Confidence Interval
Intercept	0.05	(0.01, 0.19)^a^	3.52	(0.48, 26.23)
Distance to the nearest clinic	1.00	(1.00, 1.00)^a^	1.00	(1.00, 1.00)
Rank of nearest clinic (vs. 1)				
2	0.25	(0.17, 0.38)^a^	0.46	(0.30, 0.71)^a^
3	0.17	(0.09, 0.30)^a^	0.15	(0.08, 0.30)^a^
4	0.01	(0.00, 0.03)^a^	0.09	(0.04, 0.17)^a^
Assets (vs. score 1)				
score 2	1.05	(0.66, 1.65)	0.67	(0.42, 1.04)
score 3	1.05	(0.56, 1.89)	0.56	(0.28, 1.05)
score 4	2.17	(1.33, 3.54)^a^	1.01	(0.58, 1.74)
score 5	1.15	(0.58, 2.20)	2.11	(1.16, 3.86)^a^
Years of education	0.93	(0.87, 1.00)	1.07	(0.99, 1.16)
Age	1.05	(1.01, 1.08)^a^	0.99	(0.95, 1.03)
Certainly or likely HIV positive (self-reported)	1.47	(0.85, 2.51)	1.96	(1.03, 3.71)^a^
In good health (self-reported)	0.87	(0.59, 1.31)	0.82	(0.55, 1.23)
Married (vs. unmarried)				
To a migrant	1.71	(0.89, 3.43)	0.82	(0.44, 1.53)
To a non-migrant	1.69	(0.91, 3.28)	1.04	(0.60, 1.84)
Lifetime number of pregnancies	0.93	(0.84, 1.04)	0.96	(0.86, 1.07)
Works outside subsistence agriculture	0.67	(0.46, 0.96)^a^	1.13	(0.79, 1.63)
Has a religion affiliation	0.67	(0.33, 1.45)	1.04	(0.55, 2.04)
Number of clinics within 10 *km*	1.28	(1.13, 1.45)^a^	0.93	(0.72, 1.20)

^a^significant at 0.05 level

## Discussion

While, not surprisingly, most women used the clinics that were nearest to their residences, a sizeable fraction of the sample did not. We tested five hypotheses in order to understand the underlying driving factors of clinic bypassing. The first two hypotheses, on proximity (distance to clinic) and service quality/reputation were supported by both exploratory and regression analyses, which also conform to the conclusions drawn by previous studies in Western settings (e.g. [6, 14, 16]). In particular, the district-level clinics attracted not only patients from nearby villages or from within the same district but also were used by the patients from other districts. Overall, the five district clinics (out of sixty-two included in this study) were used for prenatal care by a quarter of respondents. This disproportionate utilization of district clinics, often at the expense of more closely located facilities, conforms to the central place theory [19] that larger regional health units (district clinics in this case) share parts of the market area of the surrounding smaller units (clinics with lower rank in this case). The overall utilization pattern of local prenatal care can provides insights into future capacity design and resource allocation for the clinics at various hierarchies.

In the following three hypotheses, we looked at possible associations of economic conditions, education and health risks with clinic choice. In line with findings in Western scholarship (e.g. [12, 23]), we detected a positive relationship between greater affluence or higher health risks and the probability of bypassing the nearest clinic. However, in contrast to the well-documented role of education in the West (e.g. [6]), we did not find any comparable net effect of education in our study. It is important to note, however, that Western studies typically look at the effect of tertiary education; in our setting, like in many rural sub-Saharan settings, overall educational levels remain very low.

Although most previous studies highlighted the importance of distance and service quality in the hospital bypassing, few have examined the relationship between them. Applying the concept “range” of the central place theory, we found that service quality is consistently critical to the clinic choice regardless of distance to clinic. Thus, it can be considered that service quality, in addition to HIV infection, is the major concerns in the decision of clinic bypassing for the women travelling longer distances (Group 2). Interestingly, distance to nearest clinic and availability of nearby clinics mattered for clinic bypassing only when the actual travel distance was smaller than the “range” (Group 1). We should also note that, given the average travel distance to the visited clinic is about 6.3 *km*, the “range”, which we defined here using the median travel distance (i.e., 5.5 *km*), can be considered a conservative estimation of the actual distance that women would like to travel for prenatal care.

Our study has limitations. First, we do not have information about women’s experience of provider-patient interactions; clinic service quality is defined here solely on the basis of the variety of services and size and qualifications of clinic staff without accounting for other potentially relevant factors such as provider’s behaviour and other specifics of provider-client interactions. Another limitation is the lack of detailed information on women’s health risks associated with pregnancy; we used self-rated health and self-reported health HIV positive status possibility as proxies for respondent’s health. Further, although the fact that about 70% respondents visited the nearest clinic (Table 2) speaks to the validity of using Euclidean distance as a proxy for spatial access to health services, this approach is inevitably imperfect in capturing the characteristics of local topography (e.g. elevation and slope) and other physical barriers like rivers or lakes, which could have been addressed had additional ancillary, seasonally-adjusted data such as individual travel trajectories, land use and topography been available. In addition, we cannot account for potential influence of rural women’s daily activities on their facility choice and bypassing. For example, women might use a clinic which is not the closest but is on their way to shopping, work or visiting family and friends. In recent years, activity space constructed by location-aware devices using global positioning system (GPS) has been increasingly applied in the studies of dynamics of human activities [31]. Collecting such information in rural Africa in future research efforts will yield a better understanding of clinic choice.

## Conclusions

Facility choice and bypassing has long attracted attention in health research. Extensive literature has studied the experience in developed countries, particularly the U.S., largely focusing on the comparison of rural and urban hospitals or residents. Relatively little is known about healthcare seeking behaviour in poor developing regions such as sub-Saharan Africa, where efficient utilization of existing health services is particularly critical given limited health resources. To address this gap, this study investigated women’s clinic choice for prenatal care in a typical rural sub-Saharan setting, focusing on how and why some women would bypass the nearest clinic given the universal availability of that basic and free service at all health clinics.

Its limitations notwithstanding, however, the findings of this study fill an important gap in the knowledge of health facility choice in resource-limited settings and provide valuable information that can assist policy makers in improving health resource allocation and management by better accounting for clinic-bypassing behaviour.

## Abbreviations

GIS: Geographical information system
SRH: Sexual and reproductive health
U.S.: United States
